# Laboratory-Based and Point-of-Care Testing for MSSA/MRSA Detection in the Age of Whole Genome Sequencing

**DOI:** 10.3389/fmicb.2018.01437

**Published:** 2018-06-29

**Authors:** Alex van Belkum, Olivier Rochas

**Affiliations:** ^1^Data Analytics Unit, bioMérieux, La Balme-les-Grottes, France; ^2^Strategic Intelligence, Business Development Direction, bioMérieux, Marcy-l’Étoile, France

**Keywords:** *Staphylococcus aureus*, MSSA, MRSA, molecular testing, point of care, next generation sequencing, whole genome sequences

## Abstract

*Staphylococcus aureus* is an opportunistic pathogen of animals and humans that is capable of both colonizing and infecting its eukaryotic host. It is frequently detected in the clinical microbiology routine laboratory. *S. aureus* is capable of acquiring antibiotic resistance traits with ease and, given its rapid global dissemination, resistance to meticillin in *S. aureus* has received extensive coverage in the popular and medical press. The detection of meticillin-resistant versus meticillin-susceptible *S. aureus* (MRSA and MSSA) is of significant clinical importance. Detection of meticillin resistance is relatively straightforward since it is defined by a single determinant, penicillin-binding protein 2a’, which exists in a limited number of genetic variants carried on various Staphylococcal Cassette Chromosomes *mec*. Diagnosis of MRSA and MSSA has evolved significantly over the past decades and there has been a strong shift from culture-based, phenotypic methods toward molecular detection, especially given the close correlation between the presence of the *mec* genes and phenotypic resistance. This brief review summarizes the current state of affairs concerning the mostly polymerase chain reaction-mediated detection of MRSA and MSSA in either the classical laboratory setting or at the point of care. The potential diagnostic impact of the currently emerging whole genome sequencing (WGS) technology will be discussed against a background of diagnostic, surveillance, and infection control parameters. Adequate detection of MSSA and MRSA is at the basis of any subsequent, more generic antibiotic susceptibility testing, epidemiological characterization, and detection of virulence factors, whether performed with classical technology or WGS analyses.

## Introduction

Detection of infectious agents and their diseases is performed through a wide array of diagnostic methodologies. These range from *in silico* methods assessing host’ susceptibility to colonization and infection (Suh et al., 2018) to direct or indirect detection of the pathogen itself. The latter tests are collectively known as *in vitro* diagnostics (IVD) and their execution requires a qualified laboratory environment and highly educated technicians and (clinical) microbiologists. Next to the laboratory-based tests there are also more simple formats that should be safe to use outside of the laboratory by trained non-professionals at the point-of-need (PoN) or point-of-care (PoC). The more popular diagnostic tools are increasingly molecular in nature, having speed, specificity, and sensitivity superior to those of more classical, culture-based technologies. Molecular tests are based on different principles of which direct hybridization is among the most ancient. In addition, several different nucleic acid amplification technologies (NAATs) have been implemented [see Muldrew (2009) for a review]. Post-amplification processing often includes DNA fragment analysis and/or sequencing. Such tests are mostly aiming at detection and identification of disease-invoking bacterial species. Of note, primary detection and identification of micro-organisms are obvious pre-requisites to their further epidemiological characterization or research into their resistance and virulence characteristics. Complete diagnostic data sets can then be used for curing patients or for prevention of cross infection and infection control.

**FIGURE 1:**
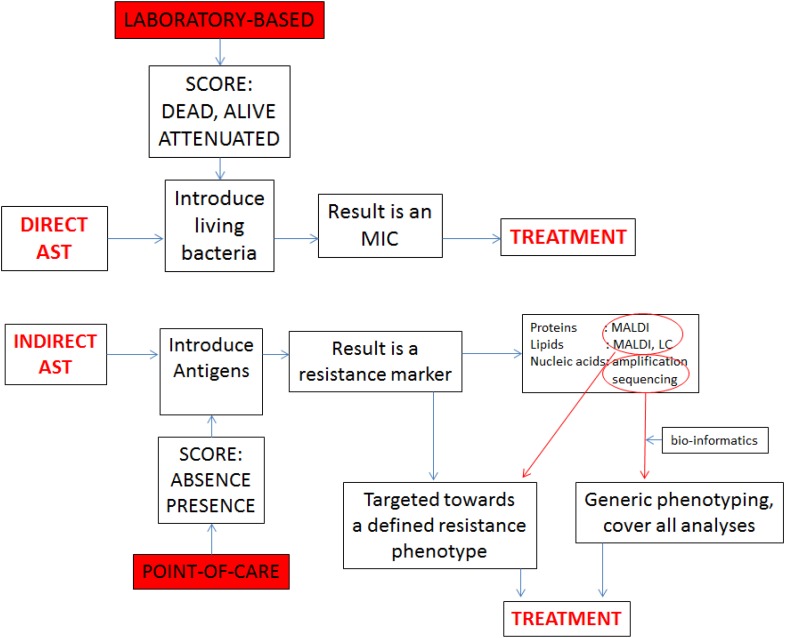
Comparison between direct AST and indirect AST, showing the difference between the laboratory based and PoC protocols. Whereas the laboratory exercise generates precise minimum inhibitory concentrations (MICs), the indirect approach generates markers that need to be associated with therapeutic modalities through bio-informatics.

In the current era of multi-to-pan antibiotic resistance, there is an increasing interest in microbial tests that detect antibiotic resistance, one of the current medical scourges (Okeke et al., 2011; Kelly et al., 2016). Although phenotypic analysis is our heritage, optimal molecular diagnosis should allow for the simultaneous detection, identification, and genetic antibiotic susceptibility testing (AST) of infectious agents. DNA sequences at the species level and at the level of resistance genes can be amplified at the same time in the same assay using the same clinical material as source (see **Figure 1** for a conceptual explanation). Indirect AST results in the detection of resistance markers and should lead to targeted treatment on the basis of the presumed activity of the product for which only the gene was detected. When innovative analytical techniques such as mass spectrometry, liquid chromatography, and nucleic acid sequencing are included advices on treatment may become more encompassing and cover-all.

Here, we will focus on the detection of meticillin resistance in the bacterial species *Staphylococcus aureus* as a model for the recent evolution of and the huge value of AST in clinical care. Meticillin-resistant *S. aureus* (MRSA) generates twice as much mortality than methicillin-susceptible *S. aureus* (MSSA) (Turnidge et al., 2009) and rapid molecular diagnostics has already been shown to reduce hospital stay and costs associated with MRSA infection (Brown and Paladino, 2010). Detecting both MSSA and MRSA is important since it guides therapeutic interventions with optimal antibiotics (Liu et al., 2011). Successful molecular diagnostic tests were developed by individual researchers (in house technology) but also by the IVD industry. In addition, quantification of the absolute number of bacterial cells in a clinical specimen is also important since different bacterial titers may be involved in colonization versus infection of human individuals. We review nucleic acid-based tests that have been made recently available for the detection of MSSA and MRSA. When such tests are correctly used they do facilitate subsequent studies into the epidemiology, evolution, and spread of both MSSA and MRSA. Detailed assessment of resistance to a wider spectrum of antimicrobial agents can be performed and implementation of enhanced infection control becomes an option.

## Culture-Based Detection of MSSA and MRSA

There is no way of discussing molecular detection of MSSA and MRSA without briefly sketching the pre- and peri-molecular diagnostic landscape. Traditionally, staphylococcal colonization, and infection were diagnosed using culture-based technologies. These either employ generic, highly fertile culture media coupled to downstream bacterial species identification or species-specific enrichment media containing *S. aureus* selective components such as elevated salt concentrations. Addition of chromogenic compounds in the medium helps to identify *S. aureus* on the basis of colony morphology and color (Perry, 2017). Further taxonomic classification and identification of *S. aureus* can be done via agglutination assays or (commercially available) biochemical reactivity (e.g., API strips). Using combinations of simple phenotypic tests has been shown to allow for the adequate distinction of MSSA and MRSA (Verroken et al., 2016; Lüthje et al., 2017; Rees and Barr, 2017; Ábrók et al., 2018). Recent immunochromatographic methods such as the BinaxNOW (Alere, Scarborough, ME, United States) and the Clearview Exact PBP2a assay (Alere, Scarborough, ME, United States) have acquired a good position in the clinical laboratory given their modest price, rapidity, and good sensitivity and specificity (e.g., Kong et al., 2014). Still, modern laboratories now consider matrix-assisted laser desorption/ionization time of flight mass spectrometry (MALDI ToF MS) as Gold Standard for staphylococcal identification (Bernardo et al., 2002; Szabados et al., 2010; Zhu et al., 2015). Distinguishing MRSA from MSSA using MALDI ToF MS is controversial with positive reports (Ueda et al., 2015; Rhoads et al., 2016; Sogawa et al., 2017) alternating with more negative ones (Du et al., 2002; Goldstein et al., 2013). Recent papers document the successful distinction between MSSA and MRSA, even including characterization of different MRSA clones (Østergaard et al., 2015; Zhang et al., 2015; Camoez et al., 2016). The main issue with these studies is that MALDI TOF MS detects proteins and that it is claimed that the *mec*A protein is produced in small amounts, often impossible to detect even by targeted mass spectrometry methods. So there is a significant risk that MALDI TOF MS will detect surrogate markers that also distinguish MRSA from MSSA. These may be markers of clonality rather than methicillin resistance and for this reason the collection of (preferably epidemiologically non-related) strains is of key importance. However, the overall impression is that MALDI ToF MS can be useful in the field of bacterial epidemiology but it will certainly not provide a universal “typing” solution. Advanced MS methods, including for instance electron spray ionization (ESI) MS, may bring more universal solutions but these methods are too cumbersome, too time-consuming and too expensive at this stage (Charretier et al., 2015). Although MS essentially provides molecular testing, albeit at the protein level, NAATs still provide the best tool for distinguishing MSSA and MRSA.

## Short Introduction into Molecular Technologies

Using polymerase chain reaction (PCR), nucleic acid sequence-based amplification (NASBA), recombinase polymerase amplification (RPA), loop-mediated isothermal amplification (LAMP) and other systems, minute amounts of DNA can be amplified and detected using a variety of technological formats (Almassian et al., 2013; Yan et al., 2014). All of these methods have been shown to be useful in the detection of infectious agents and both PCR and several non-PCR tests have been commercialized (e.g., the LAMP-based Eazyplex test, see Henares et al., 2017). New methods surface regularly (e.g., those methods employing the specificity, sensitivity of enzymes involved in CRISP*Cas-*mediated bacterial immunity to bacteriophages, see Gootenberg et al., 2017, 2018, for more details), several of them allowing genetic AST, and this import of new testing formats including their automatization will continue in the years to come.

There is a set of technologies that will undoubtedly have a huge impact on microbial detection and characterization: next generation sequencing (NGS) leading to the elucidation of the primary structure of complete bacterial chromosome sequences. Multiple elegant whole genome sequencing (WGS) NGS technologies have been developed three of which are currently commercialized and well-accessible to the diagnostic laboratory. Companies such as Illumina (San Diego, CA, United States), PacBio (Menlo Park, CA, United States), and Oxford Nanopore (Oxford, United Kingdom) provide exemplary methodologies suited for WGS. Further technical and usage detail on these methods will not be provided here but can be easily accessed through various recent reviews (Miyamoto et al., 2014; Quainoo et al., 2017; Rossen et al., 2017). NGS will find its way into the clinical microbiology routine laboratory over the years to come where it will fill in important niches in the rapid detection of pathogens and their epidemiological, antibiotic resistance, and virulence characteristics, possibly directly from clinical specimens. NGS will allow parallel sequencing of host DNA and define the host’s susceptibility to certain diseases. High throughput sequencing of RNA will allow for more precise expression monitoring via transcriptomics.

Realistically speaking though, we currently dispose of two main techniques for distinction between MSSA and MRSA: those targeting specific diagnostic signature sequences and those that characterize entire chromosomes and then depend on bio-informatic analyses to highlight the presence of the same sequence motifs used by the specific methods (**Figure 1**).

## Targeted Genetic Detection of MSSA and MRSA

It needs to be realized that for the molecular detection of MRSA there should be a differentiation between screening tests (for carriage) and hard-core diagnostic tests for infection (Osiecki, 2010; Trouillet-Assant et al., 2013). Both tests have different requirements for sensitivity, specificity, costs, and speed. Screening may not need high-speed but must be focused on specific detection of high-rate carriers (Bode et al., 2010). A major hurdle to developing molecular MRSA-specific tests is the fact that the gene encoding meticillin resistance occurs in other staphylococcal species as well. A solution to this issue is the inclusion of species-specific assays in the amplification reaction (e.g., targeting the *nuc* or *fem*A genes). The second hurdle is that the *mec* gene is present in four variants (*mec*A, *mec*C, and the more recently discovered *mec*B (Gómez-Sanz et al., 2015; Becker et al., 2018) and *mec*D (Schwendener et al., 2017)) and these reside in a growing number of genetic islands (Kolenda et al., 2017); *mec* genes are embedded in various Staphylococcal Cassette Chromosome *mec* (SCC*mec*) for which more than 10 different types have already been identified (Hill-Cawthorne et al., 2014; Kaya et al., 2018). Molecular tests for rapid discrimination of SCC*mec* types continue to be developed though their use is mostly of epidemiological rather than clinical value (Brukner et al., 2013). The *mec*C variant, mostly found in livestock associated MRSA and sharing about 70% sequence homology with *mec*A, was discovered as recently as 2011 (García-Álvarez et al., 2011). Finally, rapidity of a test can be affected by whether or not a test is (semi-)quantitative and performed in real-time or not (Verhoeven et al., 2012). The need for real-time testing differs per clinical application but in case of sepsis detection, for instance, speed is of utmost importance (Frye et al., 2012). Modern MRSA detection obviously is part of multiplexed, syndrome-oriented diagnostic testing (Blaschke et al., 2012; Ramanan et al., 2017).

A variety of experimental testing formats has been suggested for targeted MRSA detection but most of which have not reached the diagnostic market (yet). **Table 1** reviews the status of a significant number of current PCR tests and highlights a domain of importance: the next generation routine-applicable tests may very well-originate from this pool of potentially high throughput tools.

**Table 1:** Global review of future and commercial PCR tests for meticillin-resistant and -susceptible strains of *Staphylococcus aureus*.

Company	Status	Concise product description	Duration of test
Abacus Diagnostica, Finland	In development	Rapid DNA testing with proprietary GenomEra CDX-technology for identification of MRSA	50 min
AdvanDx, United States	FDA approved	*Staphylococcus* QuickFISH filter *in situ* hybridization test for positive blood culture liquid	20 min
Akonni Biosystems, United States	In development	TruArray MRSA, qualitative test for detection of SA and MRSA	Non-specified
Atlas Genetics, United Kingdom	In development	Mixed technology linking NAT and immunology for MRSA, Dual MRSA/MSSA	0.5 h
Autoi/mmun Diagnostika, Germany	CE certified	Automated AID Scanner, line probe Western blot probe assay after PCR amplification, 100 strips per hour	4 h
Biocartis, Belgium	In development	Idylla platform for multiplex real-time PCR assay for rapid detection of bloodstream infections	2 h
BioFire, United States	FDA approved, new tests in development	FDA approved syndromic panels for respiratory, gastro-intestinal, and meningitis/encephalitis associated pathogens; the BCID test also covers *mec*A. Sample in—result out strategy	1 h
BD, United States	FDA approved, new tests in development	Platform BD Max. MRSA + MSSA + *mec*A test	<3 h
Cepheid, United States (acquired by Danaher)	FDA approved for HAI with MRSA/SA	Validated for positive blood culture. Xpert test format. MRSA, SA Nasal Complete, MRSA/SA SSTI, MRSA/SA BC	2 h
Coyote Biosci, United States, China	In development	Platform Mini 8 RT PCR; throat swab/Blood sample—MRSA	10–30 min
Curetis AG, Germany	CE marked, precise status not very clear	Platform Univero; >100 pathogens and resistance genes, P55 Application focuses on pneumonia, 21 pathogens, and 19 resistance markers, 40-plex. i60 ITI Application Cartridge (23 organisms and 19 resistance genes)	4–5 h
DXna, United States	CE marked	GeneSTAT portable RT PCR platform, MRSA/MRCoNS in development for 2017	1 h
Epoch Biosciences, Elitech Group	FDA approved	Triplex Real Time Amplification tests using minor groove binding DNA probes	1 h
Genesig	RUO	Quantitative PCR for various targets among which MRSA; 16 samples per run	90–120 min
GenMark, United States	In development	Platform ePlex. Electronic sensor technology, DNA hybridization, and electrochemical detection	4 h
Genspeed, Austria	In development	Straightforward PCR with hybridization confirmation, combination of microfluidics, miniaturized opto-electronics, and automation	100 min
GFC Diagnostics	In development	Microscreen enzymatic-colorigenic DNA hybridization test on Safetube device	Non-specified
Great Basin Scientific, United States	Early stage	Whole blood, multiplexed nucleic-acid based assay using an opto-fluidic device; announced for 2021	Non-specified
Grenier Bio-One, United States	CE marked, not FDA cleared	PCR-based chip-probe Genspeed platform. Genspeed MRSA distinguishes MRSA/MRSE or *mec*A/C positive *S. haemolyticus*	1.5 h
Hain, Germany	CE marked for many tests	PCR/hybridization platform. GenoType, FluoroType and GenoQuick technologies, MRSA, CoNS	2.5 h
Icubate, United States	RUO	Random access multiplex PCR disposable test cassette for pathogens and resistances. Portfolio: gram + MSSA, *S. epidermidis*, MRSA	Non-specified
ID Biomedical, Corp., Vancouver	Early stage	Velogene rapid MRSA identification assay	2 h
Linear Diagnostics, Ltd.	In development	Detection of aligned substrate or PCR fragment via polarized light	Non-specified
Magnomics, Portugal	In development	Chip DNA extraction, amplification, and magnetic detection. Primary for veterinary application	1 h
Mobidiag, Finland	CE marked	Novodiag and Amplidiag product line. Sepsis, 60 bacterial species, 13 fungi, and *mec*A in one assay	3.5 h
Nanosphere Inc, United States	FDA cleared	DNA amplification-hybridization. Verigene BC-GP and BC-GN. Gold Nanoparticle Technology with oligo-hybridization to target DNA, narrow temperature range	2–2.5 h
Opgen, United States	Not clear	Real-time *C. difficile* and MRSA DNA testing. The Acuitas CRE Elite MDRO Gene Test detects 10 genes unique to MDRO from swabs to colonies	Non-specified
Pathogenica, Japan	RUO	Pathogenica’s DxSeq is a sequencing product. The HAI BioDetection Kit assays over 12 pathogens and 15 resistance gene families	Non-specified
Roche Molecular Systems, Germany	FDA approved	COBAS 4800 and the Liat MRSA/MSSA PCR tests	1 h

*Tests and companies may be missing and for several tests there are no peer-reviewed performance data. Diasorin and Luminex announced assays last year but the descriptions are not very precise. Same for the Spanish company Stat DX, which was recently acquired by Qiagen. Bruker and Siemens are said to be working on high throughput systems. Visibility of Chinese and Indian competitors in this field is also limited. Any mistakes are the authors’ responsibility.*

*(BC)ID, (blood culture) identification; CE, Certification Europeènne; CoNS, coagulase negative staphylococci; FDA, Food and Drug Administration; GP/N, gram positive/negative; HAI, hospital acquired infection; MDRO, multi-drug resistant organism; (MR)SA, (methicillin-resistant) *S. aureus*; SSTI, skin and soft tissue infection.*

Some of the technologies are worth mentioning separately given the fact that they can be considered extremely elegant from an experimental design point of view. Digital droplet PCR for instance was shown to be sensitive and rapid and it has to be realized that instruments allowing in house development of droplet-based PCR tests are already (commercially) available (Luo et al., 2017). Nanowires are attractive because of size, relatively low costs, and speed of the assay and broad applicability of the technology which, in addition, is easy to multiplex (Ibarlucea et al., 2017). Similarly, using albumin stabilized fluorescent gold nanoclusters as selective probes, MSSA and MRSA can be reliably distinguished (Chan and Chen, 2012). Sensitivity and specificity of these often still quite experimental tests are usually good and offer a positive perspective on future developments in this field (Bakthavatchalam et al., 2017). Note that **Table 1** highlights the post-PCR use of array technology, filter *in situ* hybridization, minor groove binding DNA probes, and magnetic capturing as additional clever read-out methods.

## Laboratory-Based vs. PoC Testing

Classical testing for microbial pathogens usually leads to amplification of viable cells. This requires the use of specialized laboratories where employees and the community outside of the lab are protected from infection through specific control measures. This has often blocked PoC test development and deployment. Now, with the possibility to detect pathogens by amplifying non-infectious components of such pathogens the door toward out-of-laboratory testing has been opened wide. Miniaturized tools have been developed that are based on microfluidics (Yeh et al., 2017), LAMP combined with cellulose-based nucleic acid binding paper (Bearinger et al., 2011), isothermal amplification tests (Toley et al., 2015) but also based upon labeling- and amplification-free techniques (Corrigan et al., 2013). With such technologies in mind it was established that PoC testing for MSSA and MRSA was among the priorities when remote and even disaster testing was due (Brock et al., 2010; Kost et al., 2012). Clearly, tools for bedside diagnostics are available that allow for in-department infection control and outbreak management.

In PoC testing both technical and clinical aspects are of key importance. Technical requirements are largely covered by the WHO ASSURED criteria. The acronym lists affordability, sensitivity, specificity, user-friendliness, rapidity and robustness, no need for complicated equipment, and providing solutions that can be easily delivered to end users. If all these requirement are met in a single test (which at this stage is non-existent) then clinical applicability is essentially global. However, if a test would, for example, be too expensive then application in developing economies would essentially be blocked. MRSA/MSSA PoC tests would be particularly useful for rapid assessment of (nasal) carriage for infection control, whereas screening for staphylococcal wound infection and respiratory infection would also have strong added value.

The first PoC MRSA projects have been published. Leone et al. (2013) did an intensive care-based study into the use of MSSA/MRSA detection in patients with ventilator-associated pneumonia. They showed that with a negative predictive value of 99.8% PoC testing efficiently excluded the presence of MRSA among the patients. They did warn that the reliability of this type of testing is dependent on the local prevalence of MRSA carriage. In an orthopedic readmission study it was shown that the Cepheid Xpert MRSA with its 75% sensitivity in this groups of patients with complicated problems performed quite well (Parcell and Phillips, 2014). Screening more than 10,000 patients at admission for detection of MRSA carriage was very efficient as well (Wu et al., 2017). Although the list of publications is relatively short, it is clear that detection of MSSA/MRSA at the PoC fulfills a real medical need. In case of epidemic spread of MRSA clones rapid and sensitive detection are key and in many cases the use of PoC testing allows for accelerated testing in comparison with more conventional laboratory assays. Speed really is the key to high throughput surveillance and subsequent rapid infection control.

## Genomic Detection of MSSA and MRSA

Single genomic molecules can be captured to microscopic beads, which are equipped with biotinylated probes to which streptavidin-complexed galactosidase binds and which facilitates the detection of sub-femtomolar concentrations of specific DNA molecules. This Single Molecule Array tests (developed by Quanterix Corporation, Lexington, MA, United States) has been adapted for the detection of MRSA as well (Song et al., 2013). Beyond capturing and detecting “full” genomes, there is now also the option to have a staphylococcal genome sequenced *de novo* and *in toto*. Thousands of MSSA and MRSA strains have been subjected to genome sequencing and the software that allows for post-sequencing detection of the *mec* genes is available (Gordon et al., 2014). Current and well-known software packages for such purposes include CLC Bio (Qiagen, Hilden, Germany), Seqsphere (Ridom, Münster, Germany), and Bionumerics (Applied Maths, bioMérieux, St. Martens Lathem, Belgium). Hence genomic characterization of MRSA is feasible and with the rise in sequencing directly from clinical specimens the impact of direct detection of MRSA will change significantly over the years to come (Lefterova et al., 2015). However, in order to be applicable in routine high-throughput clinical laboratories the technology needs to be quicker, less expensive with data that should be easy to interpret preferably in a (semi-)quantitative fashion. Obviously, genome sequencing provides the ultimate tool for epidemiological typing of MRSA and MSSA (Quainoo et al., 2017) and many studies where NGS has been exploited to define epidemiological patterns of spreading of MRSA have been published before (Tewhey et al., 2012; Price et al., 2013; Stone et al., 2013; Kong et al., 2016; Ward et al., 2016; Planet et al., 2017; and references therein).

## Concluding Remarks

Whereas classical detection and speciation of staphylococci has improved significantly upon the introduction of MALDI ToF MS in the diagnostic laboratory, molecular tests, mostly based on specific gene amplification, are still required for the rapid distinction between MSSA and MRSA. The availability of WGS and NGS has now opened up alternative avenues for the detection of resistance genes, the *mec*-variants included. The near future will bring (genome) sequencing and comprehensive software packages allowing for the unequivocal bio-informatic AST of MSSA and MRSA using WGS, even for non-bio-informaticians. The position of PoC testing in all of this is still poorly defined and needs to be clarified. Inclusion of additional patient data beyond laboratory results is an important additive to the optimization of PoC testing (Yoshioka et al., 2018). In conclusion, molecular testing for MRSA has been accepted by the diagnostic community and is performing well. New technology will challenge the molecular tests and there will be fierce clinical, commercial, and academic completion before full acceptation of the new wave of genomic testing formats.

## Author Contributions

AB and OR: conceived, wrote and illustrated the manuscript. Data for the table was assembled using aimed searches in relevant databases and publicly available information in a variety of corporate websites and communications between company employees and the authors. Omissions in the table are for the responsibility of the authors only.

## Conflict of Interest Statement

The authors are employees of bioMérieux, a company designing, developing, and selling diagnostic assays in the field of infectious diseases. Opinions and conclusions phrased in the current text are the author’s, not necessarily equaling the formal bioMérieux policies.
